# Immune Complexes Indirectly Suppress the Generation of Th17 Responses *In Vivo*

**DOI:** 10.1371/journal.pone.0151252

**Published:** 2016-03-15

**Authors:** Ceren Ciraci, John R. Janczy, Nidhi Jain, Stefanie Haasken, Cyntia Pecli e Silva, Claudia F. Benjamim, Jeffrey J. Sadler, Alicia K. Olivier, Yoichiro Iwakura, Dmitry M. Shayakhmetov, Fayyaz S. Sutterwala, Suzanne L. Cassel

**Affiliations:** 1 Inflammation Program, University of Iowa Carver College of Medicine, Iowa City, Iowa, United States of America; 2 Department of Molecular Biology and Genetics, Istanbul Technical University, Istanbul, Turkey; 3 Graduate Program in Immunology, University of Iowa Carver College of Medicine, Iowa City, Iowa, United States of America; 4 Department of Pathology, University of Iowa Carver College of Medicine, Iowa City, Iowa, United States of America; 5 Department of Internal Medicine, University of Iowa Carver College of Medicine, Iowa City, Iowa, United States of America; 6 Instituto de Ciências Biomédicas, Universidade Federal do Rio de Janeiro, Rio de Janeiro, Brazil; 7 Research Institute for Biomedical Sciences, Tokyo University of Science, Yamasaki 2669, Noda, Chiba, 278–0022, Japan; 8 Core Research for Evolutional Science and Technology (CREST), Japan Science and Technology Agency, Saitama, 332–0012, Japan; 9 Lowance Center for Human Immunology, Department of Medicine, Emory University, Atlanta, Georgia, United States of America; 10 Veterans Affairs Medical Center, Iowa City, Iowa, United States of America; French National Centre for Scientific Research, FRANCE

## Abstract

The precise context in which the innate immune system is activated plays a pivotal role in the subsequent instruction of CD4^+^ T helper (Th) cell responses. Th1 responses are downregulated when antigen is encountered in the presence of antigen-IgG immune complexes. To assess if Th17 responses to antigen are subject to similar influences in the presence of immune complexes we utilized an inflammatory airway disease model in which immunization of mice with Complete Freund’s Adjuvant (CFA) and ovalbumin (Ova) induces a powerful Ova-specific Th1 and Th17 response. Here we show that modification of that immunization with CFA to include IgG-Ova immune complexes results in the suppression of CFA-induced Th17 responses and a concurrent enhancement of Ova-specific Th2 responses. Furthermore, we show the mechanism by which these immune complexes suppress Th17 responses is through the enhancement of IL-10 production. In addition, the generation of Th17 responses following immunization with CFA and Ova were dependent on IL-1α but independent of NLRP3 inflammasome activation. Together these data represent a novel mechanism by which the generation of Th17 responses is regulated.

## Introduction

The CD4^+^ T cell response to an antigen is shaped by innate immune instruction reflecting the environment in which the antigen presenting cell (APC) initially encountered the antigen, including whether the antigen was associated with specific adjuvants or complexed with antibodies. Presentation of processed antigen with costimulatory molecules with precise combinations of cytokines drives the differentiation of naïve CD4^+^ T cells down specific effector lineage pathways including T helper (Th) 1, Th2, or Th17.

Circulating immune complexes are associated with both the initiation and the progression of many diseases and in particular autoimmune disorders. These IgG-immune complexes have been shown to be immunomodulatory and can regulate both innate and adaptive immune responses. Ligation of activating FcγR on macrophages by IgG-immune complexes results in marked suppression of IL-12p40 production, a cytokine that plays a crucial role in Th1 differentiation [1]. FcγR ligation also induces the production of the anti-inflammatory cytokine IL-10 [2]. Previous studies have demonstrated that antigen-IgG immune complexes are capable of augmenting Th2 responses [3, 4] in part through their ability to modulate the production of the cytokines IL-12p40 and IL-10. However, the effect of IgG-immune complexes on Th17 responses is unknown.

Th17 cells are typified by their elaboration of the proinflammatory cytokines IL-17A, IL-17F, and IL-22 and not only play a critical role in host defense against microbes but also drive the pathologic responses underlying autoimmune disorders [5]. The production of the innate immune cytokines IL-6, TGFβ, IL-1 and IL-23 is required for the induction of pathogenic Th17 responses [5, 6]. IL-1R signaling in T cells has been shown to be critical for the generation of Th17 cells as mice deficient in IL-1R1 have a defect in the generation of Th17 responses in an experimental autoimmune encephalomyelitis (EAE) model with an associated reduction in disease severity [7, 8].

IL-1α and IL-1β are related cytokines that both signal through the IL-1R1, yet the individual contribution of IL-1α versus IL-1β to the generation of Th17 responses remains unclear. Despite sharing a common downstream receptor, the upstream regulation of IL-1α and IL-1β secretion occurs via distinct mechanisms. The NLRP3 inflammasome is a multiprotein complex composed of the NLR family member NLRP3, the adaptor molecule ASC, and the cysteine protease caspase-1 [9, 10]. Caspase-1 becomes activated in response to specific NLRP3 agonists, which results in the processing of pro-IL-1β into its mature secreted form. IL-1α secretion, in contrast, can occur in an NLRP3 inflammasome-dependent or independent manner depending on the stimulus. Additionally, IL-1α can be released passively upon cell death and contribute to sterile inflammatory responses. Although IL-1α can be cleaved, unlike IL-1β, cleavage is not required for IL-1α to bind and signal through IL-1R1 [11].

In this study we demonstrate that the generation of CFA-induced Th17 responses was suppressed by antigen specific immune complexes. We show that while the CFA-induced Th17 response did require signaling through the IL-1R1, this signal was independent of NLRP3 inflammasome-driven IL-1β but was instead dependent upon IL-1α. Dendritic cells that encountered antigen in an immune complex produced enhanced IL-10 while concurrently suppressing IL-1α production. This coordinated modulation of dendritic cell IL-10 and IL-1α resulted in the inhibition of naïve CD4^+^ cell differentiation into Th17 effector cells. Taken together our data identify IgG-immune complexes as novel negative regulators of Th17 responses.

## Materials and Methods

### Mice

The generation of *Nlrp3*^-/-^, *asc*^-/-^, *caspase1*^-/-^, *Il10*^-/-^, *Il1r1*^-/-^, *Il1a*^-/-^, and *Il1b*^-/-^ mice have been described previously [12–17]. OT-II (B6.Cg-Tg(TcraTcrb)425Cbn/J) transgenic mice [18] were purchased from Jackson Laboratories (Bar Harbor, ME). Age and sex matched C57BL/6 and CD45.1 (B6Ly5.2Cr) mice were purchased from the National Cancer Institute. This study was carried out in strict accordance with the recommendations in the Guide for the Care and Use of Laboratory Animals of the National Institutes of Health. The Institutional Animal Care and Use Committee at the University of Iowa approved all protocols used in this study.

### Immune complexes

Chicken egg ovalbumin (Ova) (Grade V) was purchased from Sigma (St. Louis, MO). Goat IgG fraction to chicken egg Ova IgG was purchased from MP Biomedicals (Santa Ana, CA). IgG-Ova immune complexes were made by mixing a 10:1 excess of anti-Ova IgG:Ova at room temperature for 30 min. IgG-depleted antisera were generated by incubating goat anti-Ova IgG with protein G-agarose beads. IgG-depleted anti-Ova IgG (IgG^depl^) were mixed with Ova as above and used as a control. Fab fragments of whole anti-Ova IgG (MP Biomedicals) were generated with papain and purified using a Fab preparation kit (Thermo Scientific). Anti-Ova Fab fragments were mixed with Ova as above and used as a control.

### Stimulation of BMDCs

Bone marrow-derived dendritic cells (BMDCs) were generated as previously described [19]. Briefly, bone marrow was flushed from the femurs of mice, and cells cultured in RPMI1640 supplemented with 10% FCS, 2 mM L-glutamine, 100 U/ml penicillin G, 100 μg/ml streptomycin and 10 ng/ml GM-CSF (R&D systems, Minneapolis, MN); media was replenished on days 3 and 6. On day 7 suspension cells were harvested and contained cells that stained >70% double positive for MHC class II and CD11c. BMDCs were stimulated with 50 ng/ml LPS from *E*. *coli* serotype 0111:B4 (Invivogen, San Diego, CA) either in the presence or absence of 10 μg/ml Ova or IgG-Ova for 10 hrs. For the induction of IL-1α, IL1β and IL-18, 4 h after the addition of LPS cells were additionally stimulated with 5 mM ATP (Sigma) for 20 min; media was replaced with fresh media and cells were further incubated for another 6 h. Supernatants were collected and assayed for IL-1α, IL-1β, IL-18, IL-6, TNFα, IL-23, and IL-12 p40. Antibody pairs for the IL-1β ELISAs were from R&D Systems. Antibody pairs for IL-1α, IL-6, TNFα, IL-23, and IL-12 p40 were from eBiosciences (San Diego, CA). IL-18 ELISA antibody pairs were from MBL (Woburn, MA).

### Induction and evaluation of airway inflammation

Mice were sensitized on day 0 subcutaneously at the base of the tail with either 0.5 mg/ml CFA and Ova protein or 0.5 mg/ml CFA and IgG-Ova immune complexes; the final quantity of Ova was 20 μg in both cases. On days 15, 16, and 17 mice were anesthetized with isoflurane and challenged intranasally with 20 μg Ova in 50 μl PBS. Mediastinal lymph nodes (LNs), lungs, blood, and bronchoalveolar lavage (BAL) fluid were harvested on day 19. BAL was performed as previously described [20], red blood cells were lysed and total nucleated cell counts were obtained using a hemocytometer. Cytospin slides were prepared by H&E staining with HEMA 3 (Fisher) and numbers of neutrophils, lymphocytes, DC/Macs, and eosinophils quantified. Serum samples were collected on day 19 for measurement of OVA-specific IgG1 and IgG2c antibodies by ELISA as previously described [21]. Lungs were fixed, embedded in paraffin and 5 μM sections were stained with H&E.

### Cytokine analysis

Cells from the draining LNs were cultured with 10 μM OVA at 1–2 x 10^5^ cells/well for 72 h; supernatants were collected and analyzed by ELISA. Antibody pairs for IL-17A, IFNγ, IL-13 and IL-4 were from eBiosciences. For flow cytometric analysis of intracellular cytokines, LN cells were stimulated for 4 h with 50 ng/ml PMA and 500 ng/ml ionomycin in the presence of 3 μg/ml brefeldin A. Cells were fixed and permeabilized using Fixation/Permeabilization buffer (eBiosciences) and stained with anti-CD3, -CD4, -IFNγ (eBiosciences), and -IL-17A (BD Biosciences, San Jose, CA). Flow cytometric analysis was performed on a Becton Dickinson LSR II and data analyzed with FlowJo software (Tree Star Inc., Ashland, OR).

### T cell proliferation

CD4^+^ T cells were prepared by positive selection from spleens of OT-II transgenic mice using CD4 Miltenyi beads per the manufacturer’s instructions (Miltenyi Biotec, San Diego, CA). CD4^+^ T cells were labeled with 2.5 μM CFSE (Invitrogen) for 5–7 min at 37°C; 3 x 10^6^ cells were then transferred into CD45.1 congenic mice via tail vein injection. Mice were immunized with CFA/Ova or CFA/IgG-Ova as described above and T cell proliferation assessed by flow cytometry on an Accuri C6 flow cytometer (BD Biosciences) at day 3 post-immunization.

### Statistical analysis

Statistics were performed using an unpaired Student's two-tailed *t* test or two-way ANOVA. For BAL counts and serum antibody levels we performed a nonparametric Mann-Whitney *U*-test. *, p ≤ 0.05; **, p ≤ 0.01; ***, p ≤ 0.001.

## Results

### Antigen-IgG immune complexes suppress the development of Th1 and Th17 immune responses *in vivo*

**Fig 1:**
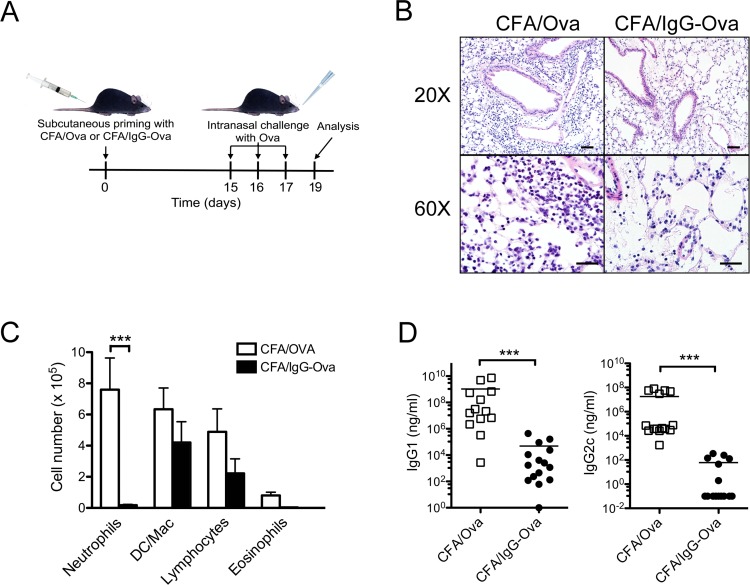
Immune complexes suppress the development of neutrophilic airway responses *in vivo*. (A) Schematic illustration of the inflammatory airway disease model; (B-D) WT mice were injected subcutaneously with either CFA/Ova or CFA/IgG-Ova on day 0; mice were then intranasally challenged with Ova on days 15, 16 and 17. 48 h after the final intranasal challenge hematoxylin and eosin lung histology sections (B) and differential cell counts in bronchoalveolar lavage fluid were analyzed (C). Representative sections from 4 mice per group are shown; upper panel bar = 50 μm; lower panel bar = 20 μm (B). Values represent the mean ± SEM of five separate experiments (n = 19 mice per group; C). (D) Ova-specific IgG1 and IgG2c levels in serum were measured by ELISA. Values represent the mean ± SEM of five separate experiments (n = 13–15 mice per group; D).

It has been shown that antigen-IgG immune complexes are capable of modulating the production of cytokines from macrophages and dendritic cells through the interaction of the immune complexes with FcγRs [1, 2]. While this has been shown to modify Th1 and Th2 responses, whether this modulation can influence the generation of Th17 adaptive immune responses is unknown. To specifically examine this question, we utilized a murine inflammatory airway disease model. Mice were immunized subcutaneously with CFA, which is a prototypical adjuvant known to induce a robust Th1 and Th17 response, along with either ovalbumin (Ova) or IgG-Ova immune complexes. Mice were subsequently challenged intranasally with Ova on days 15, 16 and 17; 48 hrs later the extent of airway inflammation was assessed by histology as well as determination of the inflammatory cell composition of the bronchoalveolar lavage (BAL) fluid (Fig 1A). As expected, mice immunized with CFA/Ova had a marked inflammatory response to intranasal challenge with Ova as evidenced by pulmonary interstitial, peribronchiolar and perivascular infiltrates of neutrophils, lymphocytes, and macrophages that were also present within alveoli (Fig 1B). Analysis of BAL fluid from CFA/Ova immunized mice was consistent with findings observed by histology (Fig 1C). In contrast, mice immunized with CFA/IgG-Ova had significantly diminished neutrophilic influx into the lungs following intranasal challenge with Ova (Fig 1B and 1C). Ovalbumin-specific IgG1 and IgG2c antibody induction was also significantly diminished in mice immunized with CFA/IgG-Ova compared to CFA/Ova (Fig 1D).

**Fig 2:**
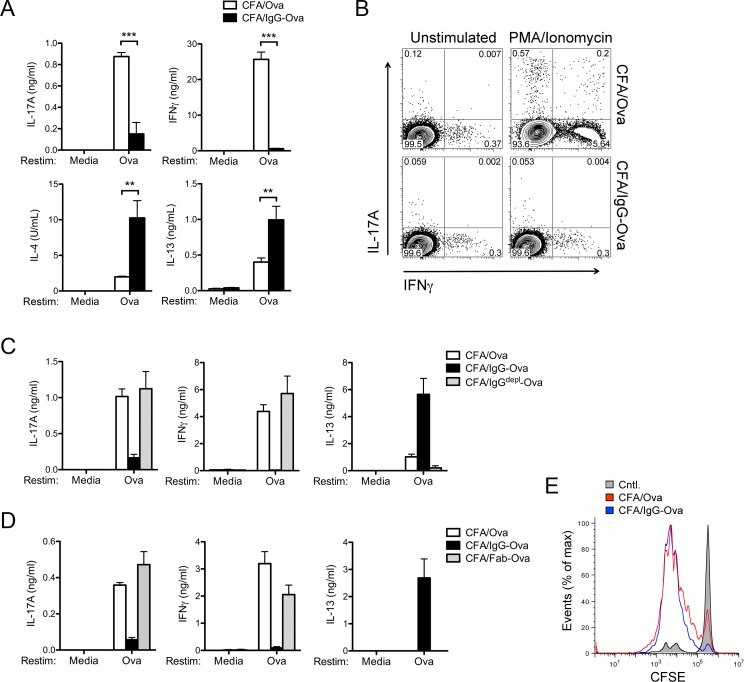
Immune complexes suppress Th1 and Th17 responses. (A) Lung draining LN were collected and restimulated *in vitro* with or without Ova (10 μM) for 72 h and cytokine levels in the supernatants analyzed. Values represent the mean ± SD and are representative of five separate experiments each with a minimum of 3 mice per group. (B) Intracellular cytokine analysis of LN cells stimulated for 4 h with PMA and ionomycin in the presence of brefeldin A. Results are representative of two independent experiments. (C, D) WT mice were injected subcutaneously with either CFA/Ova, CFA/IgG-Ova, CFA/IgG^depl^-Ova or CFA/Fab-Ova on day 0; mice were then intranasally challenged with Ova on days 15, 16 and 17. 48 h after the final intranasal challenge lung draining LN were collected and restimulated *in vitro* with or without Ova (10 μM) for 72 h and cytokine levels in the supernatants analyzed. Values represent the mean ± SD and are representative of two separate experiments each with a minimum of 2–3 mice per group. (E) CFSE labeled OT-II transgenic CD4^+^ cells were transferred into CD45.1 congenic mice, which were then immunized subcutaneously with either CFA/Ova or CFA/IgG-Ova and T cell proliferation assessed by flow cytometry 3 d later. Results are representative of two independent experiments.

To determine if the initial encounter of an antigen within an immune complex modified the subsequent adaptive immune response, we restimulated lung-draining mediastinal lymph node (LN) cells with Ova *ex vivo*. LN cells from mice immunized with CFA/Ova produced IFNγ and IL-17A upon restimulation, consistent with a strong CFA-driven Th1 and Th17 response (Fig 2A and 2B). In agreement with the diminished neutrophilic influx into the lungs of mice immunized with CFA/IgG-Ova, LN cells from these CFA/IgG-Ova treated mice also secreted significantly less IFNγ and IL-17A upon *ex vivo* restimulation with either Ova or PMA/ionomycin (Fig 2A and 2B). Surprisingly, and in contrast to the cells from CFA/Ova treated mice, LN cells from CFA/IgG-Ova immunized mice secreted IL-4 and IL-13, suggestive of the development of a Th2 effector response despite the presence of the powerful Th1/Th17 promoting and Th2 inhibiting adjuvant CFA (Fig 2A). Although the ability of dendritic cells activated *in vitro* in the presence of immune complexes to redirect a Th1 response to a Th2 response has been reported previously [3, 4], the potency of this effect *in vivo* and in the presence of such a robust Th1/Th17-inducing adjuvant as CFA was unexpected.

To control for potential nonspecific effects from the antisera we depleted IgG from antisera (IgG^depl^) and immunized mice with CFA/Ova, CFA/IgG-Ova, or CFA/IgG^depl^-Ova. LN cells from mice immunized with CFA/IgG^depl^-Ova produced similar amounts of IFNγ and IL-17A upon *ex vivo* restimulation with Ova compared to mice immunized with CFA/Ova (Fig 2C). In addition, unlike LN cells from mice immunized with CFA/IgG-Ova, LN cells from mice immunized with CFA/IgG^depl^-Ova failed to produce enhanced IL-13 (Fig 2C). To confirm that the effects of IgG-Ova immune complexes on adaptive immune responses were mediated through their Fc domain we generated Fab fragments of the anti-Ova IgG by papain digestion. LN from mice immunized with CFA/Fab-Ova produced similar levels of IFNγ, IL-17A, and IL-13 compared to LN cells from mice immunized with CFA/Ova (Fig 2D).

A possible explanation for the alteration in T cell responses could be a failure of APCs to process or present antigen when complexed with IgG. To test if APCs were capable of processing and presenting IgG-Ova and activating CD4^+^ T cells, we adoptively transferred CFSE labeled T-cell receptor (TCR) transgenic OT-II CD4^+^ T cells into mice immunized with either CFA/Ova or CFA/IgG-Ova. Dilution of CFSE showed equivalent OT-II CD4^+^ T cell proliferation in both CFA/Ova and CFA/IgG-Ova immunized mice suggesting the initial activation of CD4^+^ T cells by APCs remained intact in the presence of immune complexes (Fig 2E). That the initial activation of CD4^+^ T cells is preserved in the presence of immune complexes suggests uptake, processing, and presentation of antigen is not impacted by the presence of immune complexes. Taken together, these data demonstrate that antigen-IgG immune complexes, in an Fc dependent manner, suppress the development of Th1 and Th17 immune responses as well as having the capability of redirecting the powerful adjuvant activity of CFA to drive a Th2 immune response.

### Immune complexes modulate dendritic cell cytokine production *in vitro*

**Fig 3:**
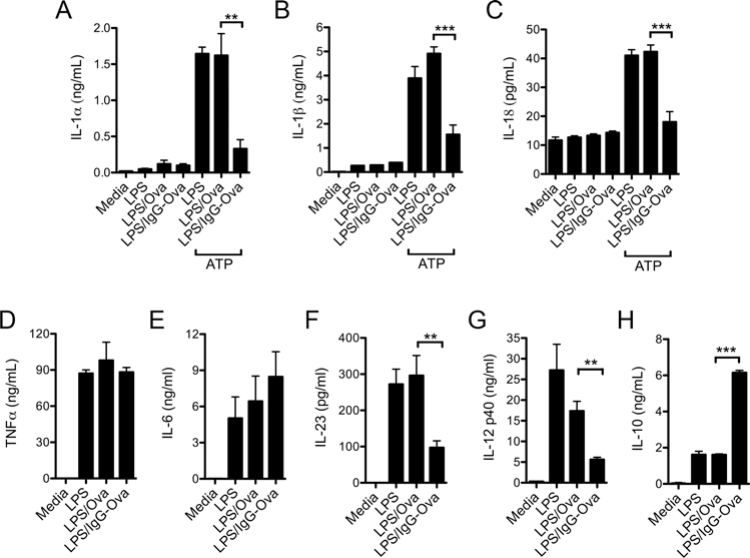
Immune complexes modulate *in vitro* cytokine production by BMDC. (A-H) BMDC from WT mice were stimulated with or without LPS (50 ng/ml) in the presence or absence of Ova or IgG-Ova immune complexes for 10 h; cytokine secretion into culture supernatants was measured by ELISA. (A-C) For the induction of IL-1α, IL1β and IL-18, 4 h after the addition of LPS cells were additionally stimulated with 5 mM ATP for 20 min; media was replaced with fresh media and cells were further incubated for another 6 h. Values represent the mean ± SEM of three independent experiments, each performed in triplicate.

Our inflammatory airway disease model demonstrated that immune complexes given *in vivo* did not impact initial T cell activation, but potently and specifically suppressed the generation of Th17 adaptive immune responses while promoting a Th2 response. Specific combinations of cytokines elicited from dendritic cells and macrophages following activation of pattern recognition receptors are required to instruct the differentiation of newly activated CD4^+^ T cells down discrete effector paths [6]. Thus, we asked if the activation of dendritic cells in the presence of immune complexes affected their production of the pivotal cytokines that drive Th17 differentiation and expansion. To assess this question, bone marrow-derived dendritic cells (BMDC) were primed with LPS in the presence of either Ova or IgG-Ova immune complexes and were then challenged with the NLRP3 inflammasome agonist ATP. As expected, stimulation of BMDC with LPS + ATP, in the absence or presence of Ova, resulted in the secretion of IL-1α, IL-1β and IL-18 [13, 22]; however, the presence of IgG-Ova immune complexes significantly inhibited IL-1α, IL-1β and IL-18 secretion (Fig 3A–3C). The production of IL-6 and TNF-α in response to LPS was not altered by the presence of immune complexes (Fig 3D and 3E); however, IL-23 production was markedly diminished in response to immune complexes (Fig 3F). Consistent with previous studies, immune complexes suppressed IL-12p40 generation, while inducing elevated IL-10 production (Fig 3G and 3H) [2]. Taken together, these data demonstrate that immune complexes modulate the production of a number of key cytokines involved in CD4^+^ T cell polarization. Of particular interest, two cytokines implicated in the differentiation and expansion of Th17 effector cells, IL-1β and IL-23, were markedly suppressed by the presence of immune complexes.

### Immune complex driven IL-10 production suppresses Th1 and Th17 responses

**Fig 4:**
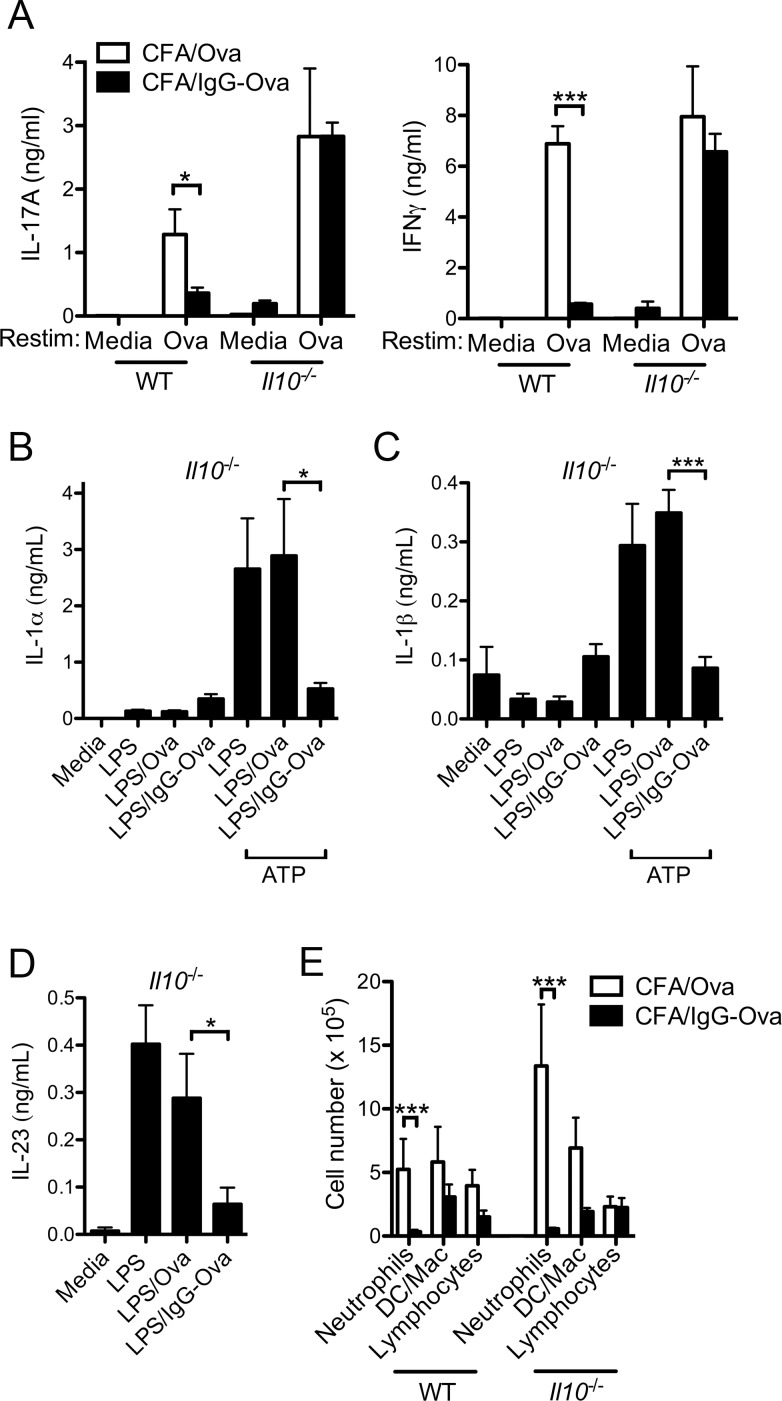
Immune complex driven IL-10 production suppresses Th1 and Th17 responses. (A) WT and *Il10*^*-/-*^ mice were injected subcutaneously with either CFA/Ova or CFA/IgG-Ova on day 0; mice were then intranasally challenged with Ova on days 15, 16 and 17. 24 h after the final intranasal challenge lung draining LN were collected and restimulated *in vitro* with or without Ova (10 μM) for 72 h and cytokine levels in the supernatants analyzed. Values represent the mean ± SD and are representative of three separate experiments each with a minimum of 3 mice per group. (B-D) BMDC from *Il10*^*-/-*^ mice were stimulated with or without LPS (50 ng/ml) in the presence or absence of Ova or IgG-Ova immune complexes for 10 h; cytokine secretion into culture supernatants was measured by ELISA. (B, C) For the induction of IL-1α and IL1β, 4 h after the addition of LPS cells were additionally stimulated with 5 mM ATP for 20 min; media was replaced with fresh media and cells were further incubated for another 6 h. Values represent the mean ± SEM of three independent experiments, each performed in triplicate. (E) WT and *Il10*^*-/-*^ mice were sensitized and challenged as described in (A), 24 h after the final intranasal challenge differential cell counts in bronchoalveolar lavage fluid were determined. Values represent the mean ± SEM of the three separate experiments (n = 6 mice per group).

Suppression of Th1 responses and augmentation of Th2 responses by *in vitro* immune complex-activated antigen presenting cells requires IL-10 [3, 4]. Whereas the influence of immune complex-elicited IL-10 on Th17 responses is unknown, IL-10 has been shown to directly suppress Th17 function as well as indirectly regulate Th17 differentiation through its effect on regulatory T cells [23, 24]. To determine if the production of IL-10 played a role in the ability of immune complexes to suppress the generation of Th17 responses, we utilized IL-10-deficient mice. Consistent with previous studies, LN cells from *Il10*^*-/-*^ mice immunized with CFA/Ova elicited enhanced production of IL-17A upon Ova restimulation compared to WT mice (Fig 4A) [25]. However, unlike WT mice, LN cells from *Il10*^*-/-*^ mice immunized with CFA/IgG-Ova failed to suppress IFNγ and IL-17A upon Ova restimulation (Fig 4A). These data suggest that immune complex-elicited IL-10 production was critical for the inhibition of CFA-driven Th17 responses.

To evaluate if IL-10 was acting indirectly by modulating dendritic cell cytokine production, the ability of IgG-Ova immune complexes to inhibit IL-1α, IL-1β, and IL-23 production was examined in BMDC from *Il10*^*-/-*^ mice. IgG-Ova immune complexes effectively suppressed the production of both IL-1α and IL-1β by IL-10-deficient BMDC in response to the NLRP3 inflammasome agonist ATP (Fig 4B and 4C). Consistent with previous data demonstrating that IL-12p40 production can be suppressed by immune complexes independently of IL-10 [26], immune complexes also suppressed the production of IL-23 in IL-10-deficient BMDC (Fig 4D). These data suggest the mechanism by which IL-10 suppresses Th17 responses is not via inhibition of BMDC cytokines necessary for Th17 differentiation.

Given the lack of suppression of IL-17A by immune complexes in IL-10-deficient mice (Fig 3A), we predicted neutrophilic airway inflammation would be similarly resistant to suppression by immune complexes in the IL-10 deficient mice. Contrary to our expectations, sensitization with CFA/IgG-Ova induced a markedly diminished neutrophilic infiltrate in the lungs of *Il10*^*-/-*^ mice following intranasal challenge with unopsonized Ova compared to sensitization with CFA/Ova (Fig 4E). These data suggest that although IL-10 is required for immune complex-driven regulation of Th17 responses, the mechanism by which immune complexes inhibit airway inflammation is discrete and independent from IL-10.

### CFA-induced Th17 responses are independent of the NLRP3 inflammasome

**Fig 5:**
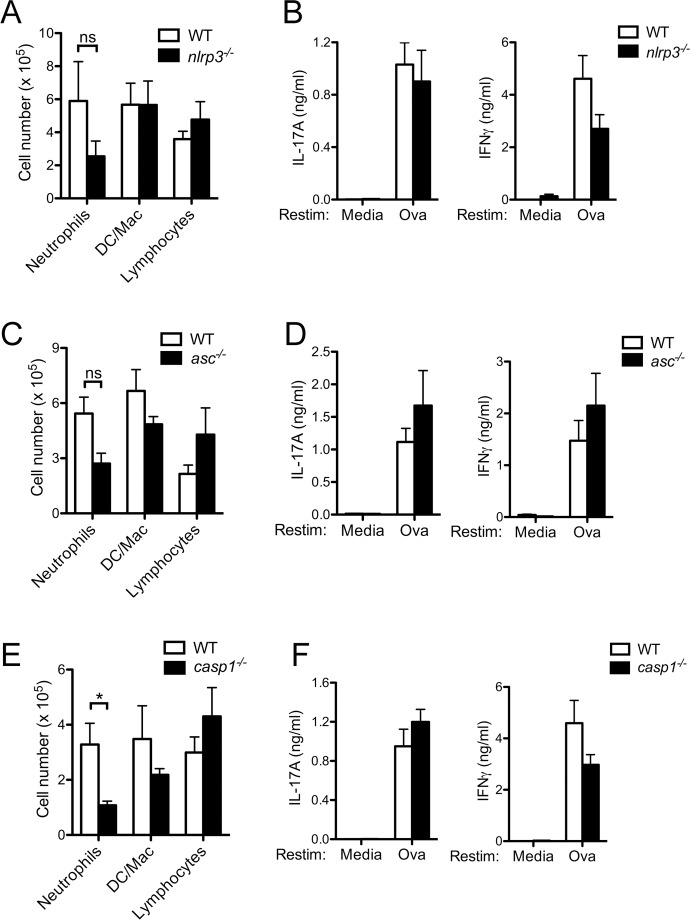
CFA-induced Th17 responses are independent of the NLRP3 inflammasome. WT, *nlrp3*^*-/-*^, *asc*^*-/-*^, and *caspase-1*^*-/-*^ mice were injected subcutaneously with CFA/Ova on day 0; mice were then intranasally challenged with Ova on days 15, 16 and 17. 24 h after the final intranasal differential cell counts in bronchoalveolar lavage fluid were determined (A, C, E). Lung draining LN were collected and restimulated *in vitro* with or without Ova (10 μM) for 72 h and cytokine levels in the supernatants analyzed (B, D, F). A, B, D, F; values represent the mean ± SEM of three separate experiments, n = 9–15 mice per group. C, E; values represent the mean ± SD and are representative of three separate experiments, n = 3–5 mice per group.

That immune complexes in *Il10*^*-/-*^ mice failed to suppress Th17 responses but maintained the ability to suppress airway inflammation suggests additional pathways regulating airway inflammation that are independent from IL-10 are modulated by immune complexes *in vivo*. The contribution of the NLRP3 inflammasome in the development of Th17 responses following immunization of an antigen in combination with the adjuvant CFA is unclear. To evaluate if the NLRP3 inflammasome was required for airway inflammation and Th17 responses in the CFA/Ova inflammatory airway model, we evaluated mice deficient in NLRP3, ASC or caspase-1. Neutrophilic influx was diminished in caspase-1-deficient mice but not in NLRP3- or ASC-deficient mice immunized with CFA/Ova and then challenged intranasally with Ova (Fig 5A, 5C and 5E) suggesting that caspase-1 driven cytokines may partially contribute to the airway inflammatory response. However, IFNγ and IL-17A production by NLRP3-, ASC- and caspase-1-deficient LN cells remained intact upon Ova restimulation (Fig 5B, 5D and 5F).

### CFA-induced Th17 responses are dependent on IL-1α signaling through the IL-1R1

**Fig 6:**
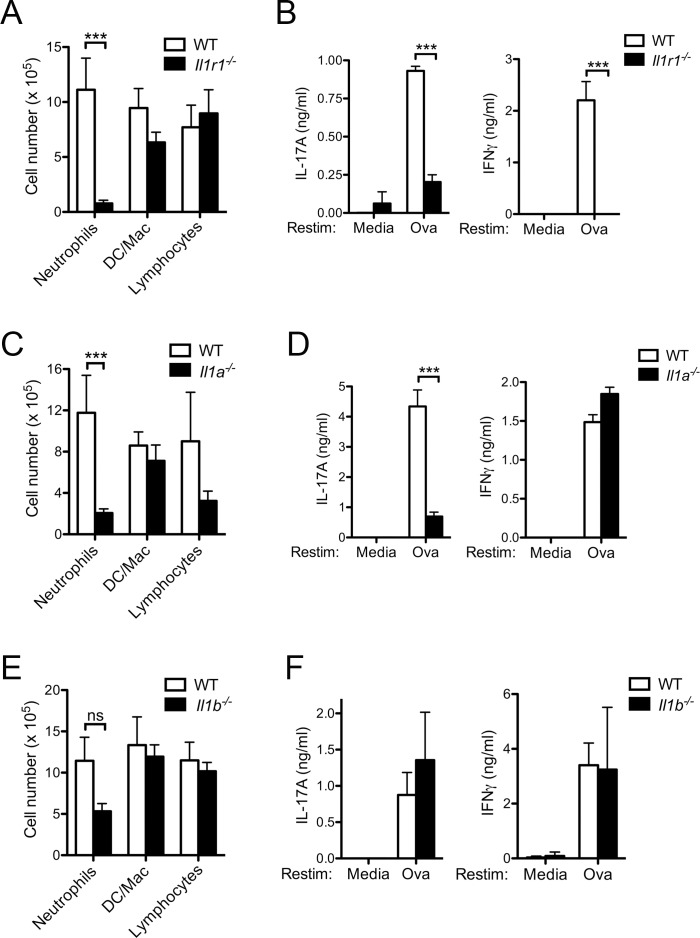
CFA-induced Th17 responses are dependent on IL-1R1 and IL-1α. WT, *Il1r1*^*-/-*^, *Il1a*^*-/-*^, and *Il1b*^*-/-*^ mice were injected subcutaneously with CFA/Ova on day 0; mice were then intranasally challenged with Ova on days 15, 16 and 17. 24 h after the final intranasal differential cell counts in bronchoalveolar lavage fluid were determined (A, C, E). Lung draining LN were collected and restimulated *in vitro* with or without Ova (10 μM) for 72 h and cytokine levels in the supernatants analyzed (B, D, F). A, C, E, F; values represent the mean ± SEM of the three separate experiments, n = 9–15 mice per group. B, D; values represent the mean ± SD and are representative of three separate experiments, n = 3–5 mice per group.

Previous studies have demonstrated that IL-1R1 signaling in CD4^+^ T cells is critical for Th17 differentiation [8]. To determine specifically the contribution of IL-1R1 signaling to airway inflammation, we induced airway inflammation in *Il1r1*^*-/-*^ mice. IL-1R1-deficient mice had markedly diminished neutrophils in the BAL compared to WT mice (Fig 6A). Consistent with this reduction in airway inflammation in the *Il1r1*^*-/-*^ mice, LN cells from *Il1r1*^*-/-*^ mice immunized with CFA/Ova had significantly diminished production of IFNγ and IL-17A upon Ova restimulation (Fig 6B), suggesting IL-1R1 signaling is required for both the T cell responses and airway inflammation induced by CFA/Ova.

Both IL-1α and IL-1β are capable of signaling through IL-1R1. To elucidate their precise involvement in Th17 responses *in vivo*, mice specifically deficient in either IL-1α or IL-1β were immunized with CFA/Ova and subsequently challenged intranasally with Ova. Markedly diminished airway inflammation and a specific defect in Th17 responses but not Th1 responses were observed in *Il1a*^*-/-*^ mice (Fig 6C and 6D). In contrast, *Il1b*^*-/-*^ mice developed similar amounts of airway inflammation compared to WT mice (Fig 6E). Similarly, IFNγ and IL-17A production by IL-1β-deficient LN cells also remained intact upon Ova restimulation (Fig 6F) suggesting signaling through IL-1R1 by IL-1α, but not IL-1β, is critical for the CFA-induced Th17 response. Taken together, these data suggest that the inflammatory airway response to intranasal challenge with Ova initiated by CFA/Ova immunization requires signaling through the IL-1R1 by IL-1α but not by IL-1β. Furthermore, the CFA-mediated Th17 response is dependent on the production of IL-1α but not IL-1β.

### Immune complexes suppress IL-1α production *in vivo*

**Fig 7:**
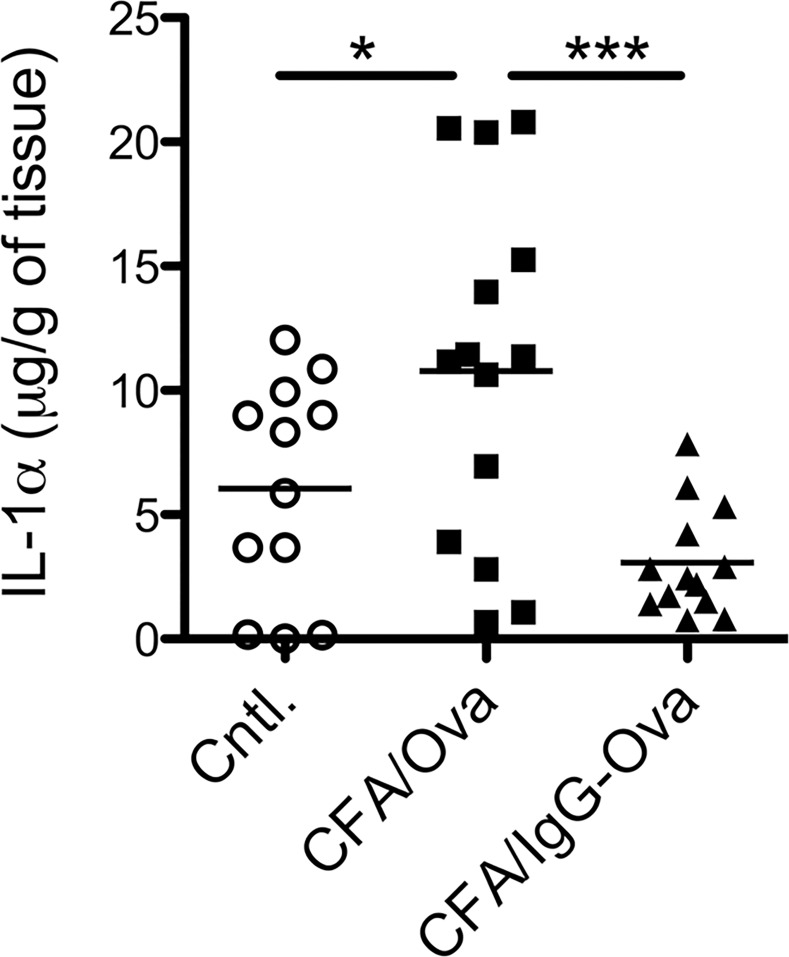
Immune complexes suppress IL-1α production *in vivo*. WT mice were injected subcutaneously with CFA/Ova or CFA/IgG-Ova; 24 h later skin at the injection site was harvested. IL-1α levels in tissue homogenates were measure by ELISA; each point represents an individual mouse.

Given the demonstrated requirement for IL-1α in CFA/Ova-induced Th17 responses and that dendritic cell IL-1α production is downregulated by IgG-immune complexes, we next asked if this suppression of IL-1α production by IgG-immune complexes occurred *in vivo*. We first examined the production of IL-1α in the skin 24 hours following immunization with either CFA/Ova or CFA/IgG-Ova. Immunization with CFA/IgG-Ova resulted in significantly less IL-1α production in the skin compared to mice immunized with CFA/Ova (Fig 7), thus demonstrating that IgG-immune complexes were also capable of inhibiting IL-1α production *in vivo*.

## Discussion

It is known the innate immune response to an antigen is dramatically altered when the antigen is associated with an adjuvant. Here we show modification of the antigen by its incorporation into a complex with antibodies also modifies the innate response and in fact can completely override the adjuvant-induced signal. This initial innate immune response is critical in shaping the ensuing CD4^+^ T cell response to the antigen. Previous studies have demonstrated that immune complexes signaling through activating FcγR on BMDC, and in particular FcγRIII, promote Th2 differentiation with a concurrent suppression of Th1 responses [3, 4]. In this study we show that immunization with CFA and IgG-Ova immune complexes resulted in the suppression of CFA-induced Th17 responses, with a concurrent enhancement of Ova-specific Th2 responses, compared to mice immunized with CFA and Ova. The mechanism by which immune complexes suppressed Th17 responses was a change in the cytokine environment in which the T cell was activated. Dendritic cells activated in the presence of immune complexes produced enhanced IL-10 production and suppressed IL-1α, favoring the development of Th2 rather than Th1 or Th17 effector cells despite the presence of the powerfully pro-inflammatory adjuvant CFA. Importantly, this finding that IgG immune complexes can wholly reprogram the Th1 and Th17 adaptive immune response typically induced by the potent pro-inflammatory adjuvant CFA was very unexpected and has significant implications for vaccine development.

The pathology associated with collagen-induced arthritis triggered by injection of collagen with CFA has been found to be independent of NLRP3 [27]. However, EAE and the associated Th17 response induced by immunization with CFA and myelin oligodendrocyte glycoprotein (MOG) is in part dependent on the NLRP3 inflammasome [28, 29]. A study by Inoue et al. noted that in EAE models using CFA containing higher concentrations of heat-killed *Mycobacterium tuberculosis*, the disease was more severe and was partially independent of the NLRP3 inflammasome [30]. Using an *in vivo* model of inflammatory airway disease we demonstrated that the CFA-driven Th17 response was dependent upon IL-1α signaling through the IL-1R1 but independent of inflammasome driven IL-1β production. This requirement for IL-1α in Th17 development, and independence from IL-1β, differs from a recent study wherein IL-1β played a dominant role [31]. The basis for these divergent findings is not clear but serves to underscore the important role of timing and route of administration of adjuvant in the development of adaptive immune responses.

Together these data demonstrate that IgG-immune complexes can suppress Th17 responses through the enhancement of dendritic cell IL-10 production. In addition, the generation of Th17 responses following immunization with CFA and Ova were dependent on IL-1α and immune complex-mediated modulation of dendritic cell IL-1α production may be an additional mechanism by which the generation of Th17 responses is regulated. Further studies will be needed to define the means by which immune complexes can be exploited to tailor an adjuvant-driven immune response to the benefit of the host.
